# Generalized Arterial Calcification of Infancy (GACI): State of the Art and Clinical Perspectives

**DOI:** 10.3390/jcdd13050184

**Published:** 2026-04-28

**Authors:** Biagio Castaldi, Giuseppe Imperatore, Elettra Pomiato, Giovanni Di Salvo

**Affiliations:** 1 Paediatric Cardiology Unit, Department of Woman’s and Child’s Health, University of Padua, 35128 Padua, Italy; elettra.pomiato@aopd.veneto.it (E.P.); giovanni.disalvo@unipd.it (G.D.S.); 2 Health Science Interdisciplinary Centre, Sant’Anna School of Medicine, 56127 Pisa, Italy; giuseppe.imperatore@santannapisa.it

**Keywords:** GACI, Generalized Arterial Calcification of Infancy, heart failure, INZ-701, targeted therapy

## Abstract

Generalized Arterial Calcification of Infancy (GACI) is a rare autosomal recessive disorder characterized by pathological calcium deposition in large and medium-sized arteries, leading to severe cardiovascular complications such as hypertension, heart failure, and stroke. The mortality rate is approximately 50% within the first six months of life if untreated. The disease is primarily caused by mutations in the ENPP1 or ABCC6 genes, resulting in a deficiency of inorganic pyrophosphate (PPi), a key inhibitor of arterial calcification. This review provides a comprehensive overview of the pathophysiology, genetic basis, and clinical features of GACI. In addition, we summarize current and emerging therapeutic strategies, including enzyme replacement therapy with recombinant ENPP1 (INZ-701), critically discussing available preclinical and early clinical evidence, as well as current limitations.

## 1. Definition

Generalized Arterial Calcification of Infancy (GACI) is a rare life-threatening genetic disorder marked by abnormal calcium deposition in the walls of large and medium-sized arteries [1,2]. This process leads to severe cardiovascular complications that significantly impact the health and survival of affected infants [2,3]. GACI is primarily caused by mutations in the *ENPP1* or *ABCC6* genes, which are essential for regulating calcium and phosphate metabolism [2,4,5]. The disease often presents in utero, with many infants showing symptoms such as hypertension, heart failure, and renal complications shortly after birth [3,6,7].

Cardiovascular complications in GACI result from diffuse arterial calcification, leading to increased vascular stiffness, hypertension, heart failure, and an elevated risk of stroke and other cardiovascular events [8,9,10].

This narrative review is based on a structured search of PubMed and major scientific databases, focusing on studies addressing the pathophysiology, genetics, and treatment of GACI.

## 2. Epidemiology

Epidemiologically, GACI is a rare disease with an estimated prevalence of approximately 1 in 200,000 live births, though estimates vary based on geographical and ethnic populations. Studies suggest that *ENPP1*-related GACI is more prevalent than *ABCC6*-related GACI, but both variants are associated with similar clinical manifestations [5]. The carrier frequency of mutations in the *ENPP1* gene is estimated to be 1 in 223 individuals, indicating that the disease may be underdiagnosed due to its rarity and the often-asymptomatic carrier status. The carrier frequency of *ABCC6* mutations varies significantly across different populations and ethnic groups and has not yet been well defined [10].

Several variants of the *ABCC6* gene display population-specific frequencies. The p.R1141X (c.3421C>T) nonsense mutation is the most prevalent pathogenic variant in European populations, accounting for approximately 29.3% of Pseudoxanthoma elasticum-associated alleles (PXE) [11,12]. Notably, the p.R1314W (c.3940C>T) missense mutation was found in homozygosity in two siblings with GACI who lacked *ENPP1* mutations, supporting *ABCC6* as a second disease locus for GACI [11]. In contrast, the p.R1339C (c.4015C>T) missense variant is the most common *ABCC6* mutation among the South African Afrikaner population, observed in 37.5% of PXE-associated alleles, likely due to a founder effect [12].

As of now, there have been around 200 documented cases in the medical literature, though the true prevalence may be higher due to undiagnosed or misdiagnosed cases [13].

GACI typically presents early, with 48% of cases diagnosed in utero or within the first month of life, and the remaining 52% presenting within the first three months [2]. However, milder forms of GACI may remain undiagnosed until later childhood or adulthood when vascular complications, such as hypertension, stroke, or organ ischemia, arise. The disease has been reported across various ethnic groups, with no clear ethnic predilection [14].

## 3. Pathophysiology

GACI is an autosomal recessive disorder characterized by a deficiency of inorganic pyrophosphate (PPi), a key inhibitor of pathological calcification [8].

*ENPP1* and *ABCC6* are involved in extracellular purine metabolism and play a central role in preventing ectopic calcification. *ENPP1* (Ectonucleotide Pyrophosphatase/Phosphodiesterase 1) hydrolyzes extracellular ATP into AMP and PPi, thereby inhibiting vascular mineralization. *ABCC6* (ATP-Binding Cassette Subfamily C Member 6), although not an enzyme, is thought to facilitate ATP release from hepatocytes, indirectly supporting PPi generation by *ENPP1* [4]. In addition, *ABCC6*-related pathways may influence the expression of tissue-nonspecific alkaline phosphatase (*TNAP*), which hydrolyzes PPi into phosphate and contributes to its reduced availability [15].

**Figure 1:**
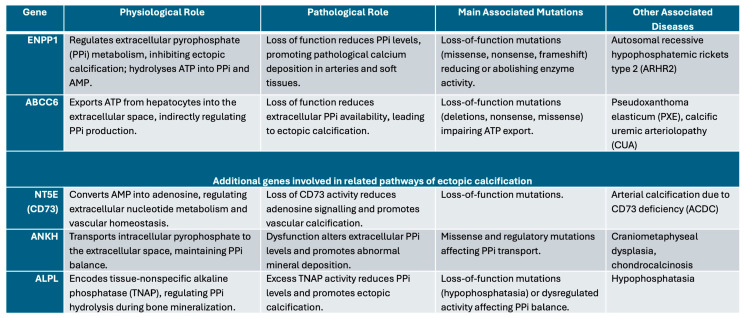
Main genes involved in Generalized Arterial Calcification of Infancy (GACI), their physiological and pathological roles, common mutations, and other associated diseases [18,19,20,21].

Mutations in *ENPP1* [4,16] (GACI type 1) or *ABCC6* [12] (GACI type 2) disrupt PPi homeostasis, leading to a relative excess of inorganic phosphate (Pi) and a shift in the PPi/Pi ratio toward calcification [1,9]. *ENPP1* mutations are typically associated with a marked reduction in PPi levels, whereas *ABCC6* mutations, although less well characterized, result in a similar pro-calcific state. Both genes regulate extracellular matrix (ECM) mineralization, and their dysfunction leads to systemic ectopic calcification (Figure 1) [1,4,9]. Experimental models, including *ENPP1*-deficient mice and zebrafish, recapitulate key disease features and provide platforms for therapeutic investigation [1].

The clinical phenotype varies according to the underlying genetic defect. *ENPP1* mutations are generally associated with severe neonatal disease and high mortality, whereas *ABCC6* mutations may present with a milder, later-onset phenotype [2,17]. In addition, compound heterozygous *ENPP1* variants may contribute to a spectrum of disease severity [9].

## 4. Pyrophosphate Deficiency as the Central Pathogenic Axis in ENPP1-Mediated GACI

Extracellular PPi is the principal endogenous inhibitor of hydroxyapatite formation, and its deficiency represents the central biochemical abnormality in *ENPP1*-mediated GACI. *ENPP1* hydrolyzes extracellular ATP into AMP and PPi; therefore, *ENPP1* deficiency should be viewed not only as a reduction in PPi availability but also as a dual defect involving impaired AMP/adenosine-mediated vascular signaling [3,4].

This distinction has therapeutic implications. While PPi analogs may partially limit mineral deposition, recombinant *ENPP1* replacement can restore upstream metabolic pathways more comprehensively, with potential effects on intimal proliferation and vascular remodeling [4].

**Figure 2:**
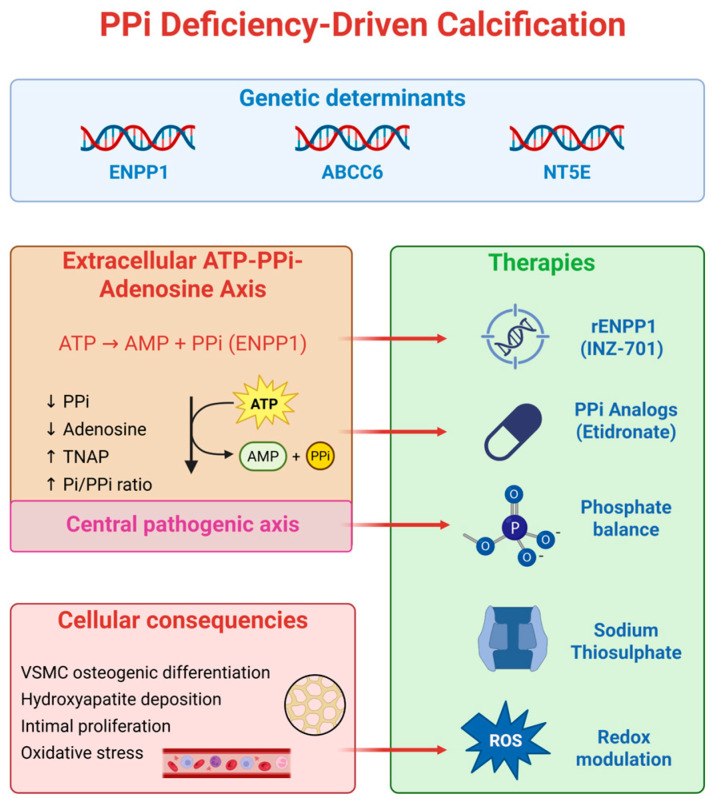
Pathophysiology and therapeutic targets in PPi deficiency–driven calcification. Genetic defects in ENPP1, ABCC6, and NT5E disrupt the extracellular ATP–PPi–adenosine axis, leading to reduced inorganic pyrophosphate (PPi), altered adenosine metabolism, and an increased Pi/PPi ratio. This imbalance promotes vascular smooth muscle cell (VSMC) osteogenic differentiation, hydroxyapatite deposition, intimal proliferation, and oxidative stress. The central pathogenic axis highlights impaired PPi generation and signaling. Therapeutic strategies aim to restore this balance through ENPP1 replacement, PPi analogs, phosphate homeostasis, sodium thiosulfate, and redox modulation.

An integrated overview of the molecular mechanisms underlying inherited arterial calcification and the main therapeutic targets is shown in Figure 2.

## 5. Clinical Presentation

**Figure 3:**
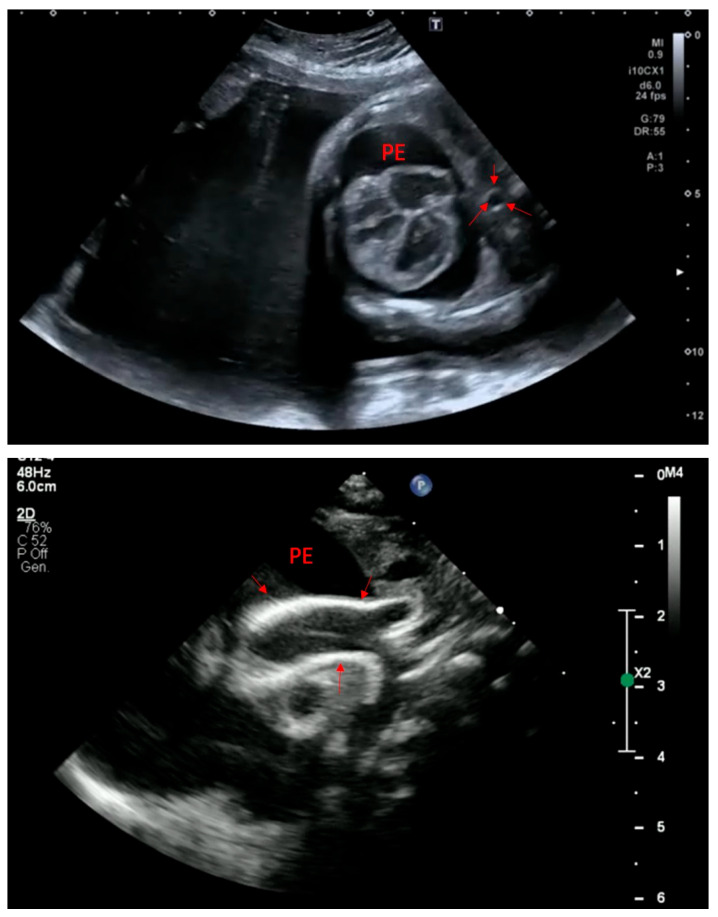
A case of neonatal GACI. On the top, fetal heart failure characterized by pericardial effusion (PE). Posteriorly, thoracic aorta showed a diffuse hyperechogenicity (arrows) Bottom: the same baby immediately after birth. Pericardial effusion was confirmed, and aortic wall brightness was much more evident (arrows).

**Figure 4:**
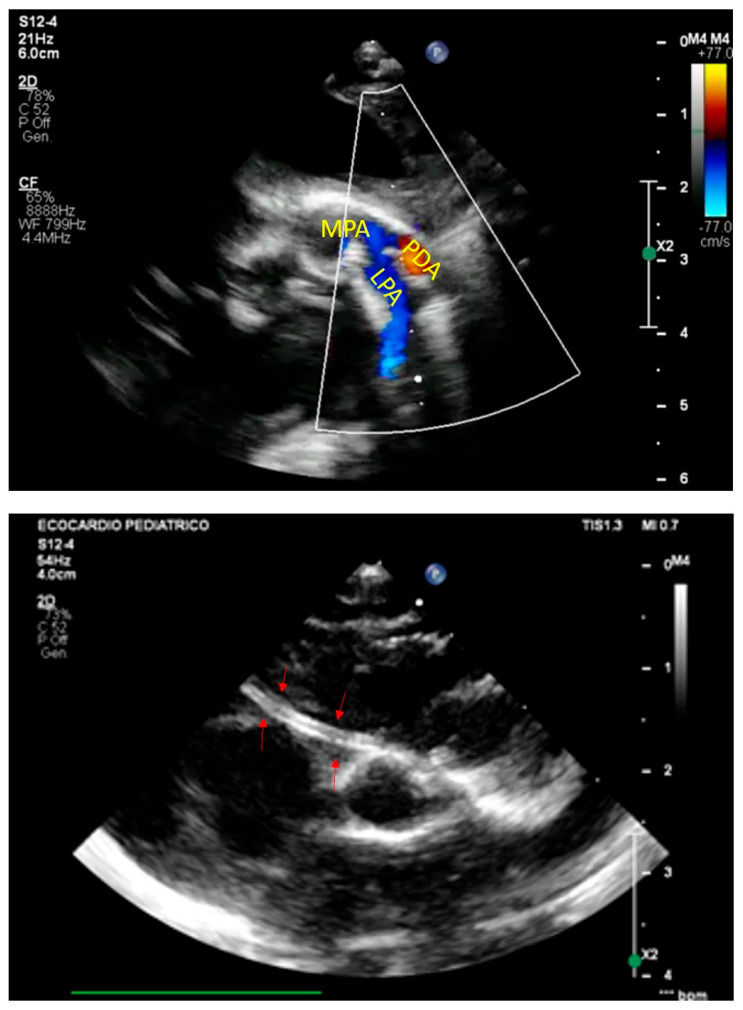
Top: arterial duct calcification (PDA) in a case of GACI. MPA: main pulmonary artery, LPA: left pulmonary artery. Bottom: diffuse calcification of right coronary artery (arrows).

The clinical presentation of GACI is highly variable, reflecting a spectrum of severity influenced by genetic mutations and residual enzyme activity. Symptoms typically manifest within the first few weeks of life, although prenatal signs such as polyhydramnios, fetal distress, and intrauterine growth restriction have been reported [6,14]. The hallmark of GACI is extensive arterial calcification, particularly affecting large and medium-sized arteries, leading to severe vascular dysfunction and early-life cardiovascular complications (Figure 3 and Figure 4) [6].

**Figure 5:**
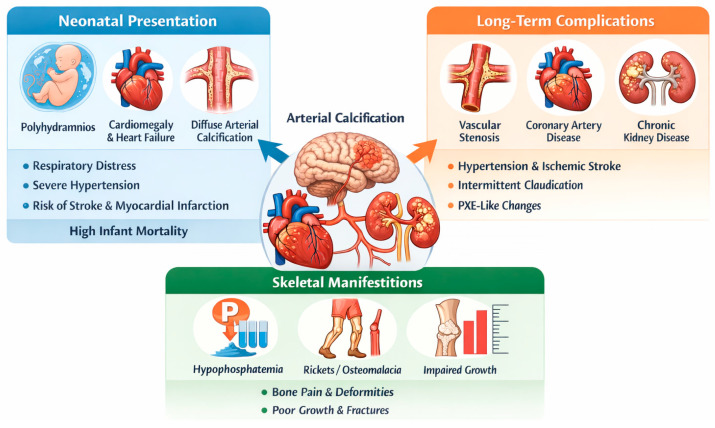
Clinical spectrum and systemic manifestations of GACI. The figure illustrates the continuum from neonatal cardiovascular presentation to long-term vascular complications, including arterial stenosis, coronary artery disease, and PXE-like features. Musculoskeletal involvement is also shown, with hypophosphatemia, rickets/osteomalacia, impaired growth, and increased fracture risk, reflecting the systemic nature of the disease.

Several key features are observed: dilated and hypertrophied ventricles, cardiomegaly, and pulmonary plethora, mild concentric ventricular hypertrophy and vascular calcifications [2,14]. Extramedullary hematopoiesis in the liver and periarticular calcifications in areas like the wrists, ankles, and hips are also common [2]. Diffuse arterial calcification affects major arteries such as the aorta, carotid, and coronary arteries [8,22]. Brain ultrasound may show dilated ventricles and leukomalacia, while abdominal ultrasound can reveal hepatosplenomegaly and calcifications in multiple organs (Figure 5) [23].

### 5.1. Neonatal and Infantile Presentation

Most affected infants present with respiratory distress, severe hypertension, and congestive heart failure due to arterial stiffening, increased afterload, and left ventricular hypertrophy. Peripheral pulse attenuation may reflect progressive arterial stenosis, predisposing to myocardial infarction or stroke in severe cases. Imaging typically reveals diffuse arterial calcifications and echocardiographic signs of cardiac dysfunction (Figure 3). Without intervention, mortality remains high within the first six months of life, mainly due to cardiopulmonary failure.

### 5.2. Later-Onset and Atypical Presentations

A subset of patients with residual *ENPP1* activity or *ABCC6*-related disease may survive beyond infancy and exhibit a less aggressive but progressive vascular phenotype [24]. In these cases, arterial calcification may stabilize or regress, but long-term complications such as renovascular hypertension, peripheral artery disease, and intermittent claudication can arise. Some individuals develop pathological arterial remodeling, resulting in intimal proliferation and stenotic lesions, further exacerbating vascular occlusions [2].

### 5.3. Cardiovascular Implications and Long-Term Sequelae

Patients surviving into adolescence or adulthood may develop a chronic vasculopathy resembling pseudoxanthoma elasticum, characterized by progressive arterial stiffness, hypertension, coronary artery disease, and an increased risk of ischemic stroke. Diffuse medial calcification leads to vascular stenosis, reduced organ perfusion, and secondary complications such as nephrovascular hypertension and chronic kidney disease.

Vascular stenosis arises from medial arterial calcification, particularly along the internal elastic lamina, resulting in vessel wall thickening and luminal narrowing without associated inflammation. Stenoses commonly involve coronary, renal, and large systemic arteries, contributing to myocardial ischemia, severe hypertension, and end-organ damage [2,17]. Renal involvement may further exacerbate hypertension and lead to chronic kidney disease (CKD) [17].

Cardiovascular involvement is a major determinant of morbidity, with arterial stiffening increasing afterload and contributing to left ventricular hypertrophy, heart failure, and reduced cardiac output, particularly when the aortic arch and coronary arteries are affected [2,7]. Cerebral vascular involvement may result in ischemic or hemorrhagic events, potentially leading to permanent neurological damage [2,25].

Although early mortality remains high, some patients survive into adulthood but remain at risk of progressive vascular disease, including persistent hypertension, arteriosclerosis, and recurrent ischemic events. In these individuals, extra-vascular manifestations resembling PXE may also occur, involving the skin, eyes, and cardiovascular system [2,7,26].

## 6. Diagnosis

### 6.1. Prenatal Diagnosis

Prenatal diagnosis of GACI relies on the detection of characteristic ultrasound markers, which are crucial for early identification of this rare disorder. Increased echogenicity of arterial vessels, notably the aorta, pulmonary arteries, coronary arteries, and renal arteries, is often observed as early as the second trimester, especially after 20 weeks of gestation [2,27]. These vessels appear markedly brighter on ultrasound due to the calcium deposits in the arterial walls, which impede normal blood flow and lead to structural abnormalities. Such ultrasound findings are highly suggestive of GACI, and their presence warrants further investigation through molecular genetic testing to confirm the diagnosis [28].

Additional ultrasound indicators include fetal hydrops, and pericardial effusion, all of which may be present as part of the disease’s vascular compromise [28]. In some cases, these features are accompanied by placental insufficiency and intrauterine growth restriction, which may serve as early clinical clues [3]. Ultrasounds play a pivotal role in detecting these signs, and should be performed periodically throughout the pregnancy when GACI is suspected. When calcifications are identified, a tempestive prenatal counseling is recommended to inform parents about post-natal management and clinical implications of the disease. After birth, genetic test can be performed to confirm the diagnosis. However, medical treatment to reduce calcium deposition, to manage hypertension, and for cardiovascular support, can be properly initiated [22]. In particular, studies have suggested that bisphosphonates may be used to reduce calcification, although their efficacy in GACI is still being investigated [22,28]. A study showed that there are no statistically significant survival benefits in patients treated with bisphosphonates compared to those who were not treated, showing that despite the theoretical benefits of bisphosphonates in reducing arterial calcification and potentially slowing disease progression, they may not significantly alter the survival outcomes for these specific patients [17].

### 6.2. Calcium Score

Although echocardiography is useful for the early detection of vascular involvement in GACI, it does not allow for quantitative assessment of calcification burden. In clinical and research settings, vascular calcification is more accurately evaluated using computed tomography (CT)-based techniques, particularly through the Agatston calcium score. This method quantifies calcified plaque burden by integrating both the area and density of calcific deposits, providing a reproducible and widely validated measure of vascular calcification severity [28,29].

CT-derived calcium scoring has been extensively used in cardiovascular medicine and in populations with altered mineral metabolism, such as patients with chronic kidney disease. Importantly, studies have demonstrated that alterations in the ENPP1 pathway may directly influence calcification burden as assessed by CT. For instance, individuals carrying ENPP1 variants have been shown to exhibit significantly higher coronary artery calcium scores compared to matched controls, supporting a mechanistic link between impaired pyrophosphate metabolism and accelerated vascular mineralization [28].

These observations highlight the importance of integrating quantitative imaging modalities in the evaluation of inherited and acquired calcification disorders. In the context of GACI and related conditions, CT-based calcium scoring may represent a valuable tool not only for disease characterization but also for monitoring progression and response to emerging therapies [28,29].

### 6.3. Differential Diagnosis and Follow-Up

Several conditions may mimic GACI, including other genetic disorders of ectopic calcification such as NT5E (CD73) deficiency, pseudoxanthoma elasticum, and secondary causes of vascular calcification such as chronic kidney disease or metabolic disturbances. Differentiation relies on genetic testing, biochemical profiling, and clinical context.

Longitudinal follow-up of patients with GACI should include serial imaging to assess progression or regression of vascular calcification. Echocardiography remains useful for functional assessment, while CT-based techniques may provide quantitative evaluation over time. Biomarker monitoring, including PPi levels, alkaline phosphatase activity, and phosphate metabolism parameters, may offer additional insights into disease activity.

## 7. Prognosis

The prognosis for untreated infants with GACI is generally poor, with an elevated intrauterine death (24%). and a 55% mortality within the first six months of life [2,24]. The primary causes of death in these infants are myocardial infarction, heart failure, persistent hypertension, and multiorgan failure [7,9]. In contrast, adults with GACI generally have a better prognosis, although they face long-term progressive vascular calcification, and ultimately chronic cardiovascular complications [26,27]. Early diagnosis, combined with aggressive treatment, is critical in improving survival rates and enhancing quality of life for affected individuals.

In survivors, long-term outcomes may include persistent vascular disease, hypertension, and variable degrees of functional impairment. Data on neurodevelopmental and quality-of-life outcomes remain limited, highlighting the need for long-term follow-up studies.

## 8. Current and Emerging Treatments

Treatment options are continually evolving owing to the progress in the understanding of the disease. Currently, symptomatic therapy remains the cornerstone of management, particularly in addressing the cardiovascular complications associated with the disease, such as hypertension and heart failure [7]. Medications commonly used in the management of these symptoms include antihypertensive agents, diuretics, ACE inhibitors or angiotensin II receptor blockers (ARBs), all of which help to reduce blood pressure, alleviate cardiac strain, and improve circulation [2,28]. Aspirin may also be prescribed to prevent clot formation in patients with severe coronary artery stenosis, ensuring better blood flow [30,31].

Regular monitoring and follow-up care are essential to adjust treatments as needed and to manage emerging symptoms. These therapies, though not curative, can significantly improve the quality of life and manage the complications of the disease [32]. However, these treatments do not target the underlying cause of the disease, and their efficacy in improving long-term outcomes is limited [33].

Bisphosphonates, especially etidronate, have been used in GACI because they function as non-hydrolysable PPi analogs and may inhibit hydroxyapatite deposition within the arterial wall. However, reported clinical effects remain heterogeneous [14,22]. This variability likely reflects differences in timing of treatment, residual ENPP1 activity, severity of baseline vasculopathy, tissue distribution and hydroxyapatite-binding characteristics of different PPi analogs, and the fact that ENPP1 deficiency affects not only calcification but also AMP-dependent vascular biology [22]. Accordingly, PPi analogs may partly mitigate mineralization while incompletely addressing the broader vasculopathy of ENPP1 deficiency.

A promising advancement in the treatment of GACI is enzyme replacement therapy (ERT) with recombinant *ENPP1*, which directly addresses the biochemical deficiency at the root of the disorder. Recombinant *ENPP1* aims to restore normal PPi levels, thereby preventing the excessive calcification of arteries [10]. Animal models have demonstrated that ERT with recombinant *ENPP1* can significantly reduce arterial calcification, improve hypertension, and enhance cardiac function [1,2]. Although this therapy shows considerable promise, potential side effects include allergic reactions and infusion-related complications such as fever, rash, or anaphylaxis. These side effects are generally mild, but warrant monitoring during treatment [9,10].

**Figure 6:**
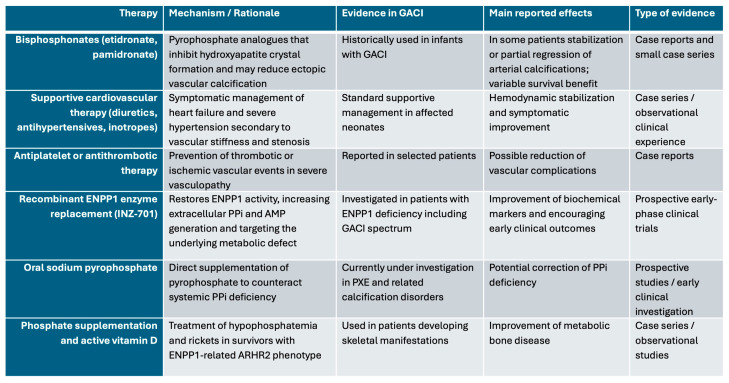
Pharmacological therapies used or investigated in GACI.

Beyond enzyme replacement therapy, several experimental strategies are being explored for GACI (Figure 6). Sodium Thiosulfate (STS) has emerged as a potential adjunctive therapy, but its mechanism of action extends beyond a simple calcium-chelating effect. Experimental evidence from cell culture and animal models suggests that STS also acts as a sulphur donor, modulating redox-sensitive pathways and influencing vascular smooth muscle cell (VSMC) phenotype. STS and hydrogen sulphide (H_2_S)-related signaling have been shown to attenuate phosphate-induced vascular calcification and inhibit osteogenic transdifferentiation of VSMCs, likely through effects on oxidative stress, alkaline phosphatase activity, and intracellular signaling pathways [26,34].

These findings support a more complex biological role for STS in vascular calcification, integrating physicochemical and cell-mediated mechanisms. However, current evidence remains largely preclinical, and clinical experience in GACI is limited (Figure 6). Moreover, treatment may be associated with adverse effects such as nausea, vomiting, and metabolic acidosis, which may restrict its long-term use and require careful monitoring.

### 8.1. Clinical Perspective: Neonatal Phosphate and Vitamin D Supplementation in Infants with Possible Inherited Calcification Susceptibility

In contemporary neonatal practice, phosphate supplementation and vitamin D administration are often necessary, particularly in preterm infants at risk of metabolic bone disease. Nevertheless, in neonates with unexplained arterial echogenicity or calcification, severe hypertension, cardiac dysfunction, fetal hydrops, or a family history suggestive of inherited ectopic calcification, clinicians should be cautious about unnecessary supraphysiologic mineral loading [17]. While routine neonatal supplementation remains appropriate when clinically indicated, excessive phosphate exposure and high vitamin D intake could theoretically aggravate calcification in genetically susceptible infants by further shifting the local pro-mineralization milieu [29]. In these settings, early biochemical and genetic evaluation should be considered.

### 8.2. Upcoming Solutions

Owing to preclinical progress in the understanding of the disease, new molecules and targeted therapies for GACI are emerging [33]. However, more studies are needed to fully assess their long-term efficacy [34].

**Figure 7:**
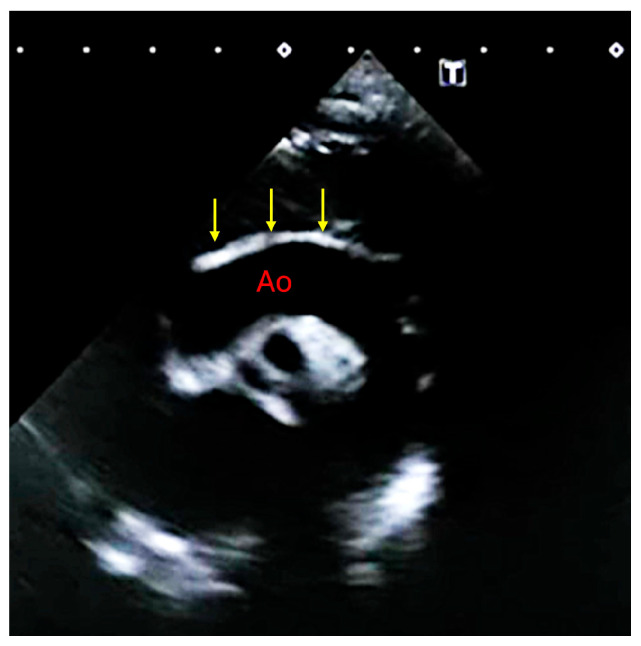
Compassionate use of INZ-701 in a newborn with severe GACI. Six months after initiation of therapy, the aortic calcification score markedly improved (see Figure 3 for neonatal presentation). The aortic (Ao) wall appears thinner and less hyperechoic compared with baseline at birth.

INZ-701 is a new ERT for clinical trials about GACI and other genetic disorders related to *ENPP1* deficiency. Developed by Inozyme Pharma, this therapy targets a central defect in the PPi-Adenosine Pathway, offering potential to treat multiple rare diseases caused by disruptions in this pathway (Figure 7).

Chemically, INZ-701 is a recombinant enzyme that combines the active part of the *ENPP1* enzyme with a fragment of a human antibody (Fc), allowing it to circulate in the bloodstream and convert ATP into inorganic pyrophosphate (PPi) and AMP [10,35]. By restoring PPi levels, INZ-701 helps prevent pathological calcium deposition in arteries and tissues, reducing the risk of severe cardiovascular complications like those seen in GACI [36]. Administered via subcutaneous injections, INZ-701 offers sustained release and a long half-life, enabling less frequent dosing compared to already known therapies. Early clinical trials, namely NCT04686175, have demonstrated that the drug is well tolerated, with no serious adverse events reported. Potential side effects include allergic reactions, infusion-related reactions, and immune responses, which are generally mild and comparable to those observed with other ERTs. In clinical trials, INZ-701 has shown significant benefits. Animal studies investigating the effects of INZ-701 on neointimal proliferation in both wild-type and *ENPP1*-deficient mice have reported improvements in PPi levels, reductions in vascular calcification, and enhanced cardiovascular health. Notable results from Boyce et al. [2] indicated that INZ-701 reduces calcification and mortality, while in a study from Ferreira et al. [1] INZ-701 prevented osteomalacia and nephrocalcinosis.

However, INZ-701 has some limitations. It requires regular injections, and a degree of variability in patient response due to the development of anti-drug antibodies has been documented [37]. Additionally, its long-term effects are still under investigation, and further research is needed to understand its efficacy across different patient populations.

### 8.3. Ongoing Clinical Trials on INZ-701

**Figure 8:**
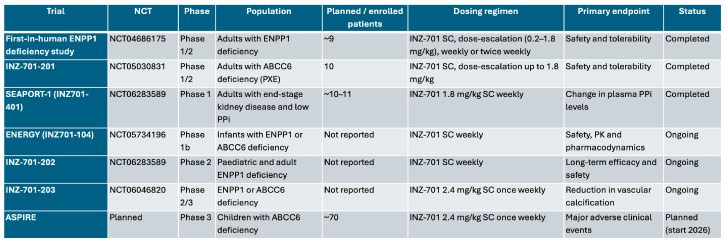
Ongoing and completed clinical trials evaluating INZ-701.

Several clinical trials are currently evaluating INZ-701 across different patient populations. Early-phase studies, including SEAPORT 1 and the ENERGY programme (phases 1b–3), have demonstrated sustained increases in circulating PPi levels and favorable safety profiles. Ongoing and planned studies, such as ADAPT, ASPIRE, and the INZ-701-201/202/203 trials, aim to assess long-term safety, pharmacodynamics, and clinically meaningful outcomes, including cardiovascular events and functional status (Figure 8).

The ENERGY study (NCT05734196) is a Phase 1b open-label trial assessing the safety, pharmacokinetics, and pharmacodynamics of INZ-701, a recombinant *ENPP1* enzyme replacement therapy, in infants with *ENPP1* or *ABCC6* deficiency. This study, along with its subsequent phases (ENERGY 2 and 3), aims to evaluate the drug’s effectiveness in treating GACI by reducing arterial calcification, improving cardiovascular function, and enhancing overall health. Key outcomes include better vascular health, reduced calcification, and improved quality of life. Preliminary results show promising benefits in managing symptoms and preventing disease progression, with ongoing research focused on long-term safety and efficacy.

The ASPIRE pivotal trial is a planned RCT designed to evaluate the safety and efficacy of INZ-701 in children with *ABCC6* deficiency, supported by both the FDA and EMA and reviewed and received preliminary support from EU regulators. Set to begin in early 2026, the trial will focus on severe disease complications in children, enrolling 70 patients aged from infancy to 18 years, including those with biallelic and monoallelic *ABCC6* mutations. The primary endpoint will evaluate major adverse clinical events over a two-year treatment period.

The INZ-701-201 trial (NCT05030831, phase 1/2) focused on assessing the safety, tolerability, and pharmacodynamics of INZ-701 in patients with *ENPP1* deficiency, particularly its effects on arterial calcification and cardiovascular health. Following this, the INZ-701-202 trial (NCT06283589, phase 2) expanded the scope to evaluate the long-term efficacy and safety of the drug in both pediatric and adult patients, aiming to demonstrate improvements in cardiovascular function. Finally, the ongoing INZ-701-203 trial (NCT06046820, phase 2/3) is designed to further explore the effectiveness of INZ-701 in treating GACI due to *ENPP1* or *ABCC6* deficiency, with a focus on long-term reductions in vascular calcification and enhancements in quality of life and functional outcomes.

## 9. Conclusions

Generalized Arterial Calcification of Infancy is a rare, complex, and often devastating disorder that requires a comprehensive understanding of its genetic and pathophysiological mechanisms to guide effective treatment strategies. Although current treatment options remain limited, primarily focusing on symptom management, recent breakthroughs in ERT, such as INZ-701, provide new hope for improving patient outcomes. This innovative therapy targets the root cause of the disease by replenishing deficient PPi levels, thus halting the progression of pathological calcification.

While early clinical trial results have shown promise, challenges persist in the management of GACI due to its rarity, the wide variability in its clinical manifestations, and the need for robust, long-term safety and efficacy data. As the clinical application of INZ-701 is still being refined, additional research is necessary to determine the optimal dosing, timing, and patient selection for its use. The ongoing development of INZ-701, along with other novel therapies targeting the underlying genetic defects, highlights the importance of continued research in this field.

These therapies offer the potential not only to halt the progression of vascular calcification but also to improve the overall quality of life for patients with GACI. Given the severe nature of the disease and its impact on multiple organ systems, it is crucial to pursue further investigation into these treatments to maximize their benefits. Ultimately, ongoing clinical trials and research will be vital in defining the role of INZ-701 and other therapies in the long-term management of GACI, ensuring that affected patients receive the most effective and personalized care possible.

## Data Availability

No new data were created or analyzed in this study.
